# LncRNA DLEU1 contributes to colorectal cancer progression via activation of KPNA3

**DOI:** 10.1186/s12943-018-0873-2

**Published:** 2018-08-11

**Authors:** Tianyou Liu, Zhiyang Han, Huanyu Li, Yuekun Zhu, Ziquan Sun, Anlong Zhu

**Affiliations:** 1 Department of General Surgery, The First Affiliated Hospital of Harbin Medical University, #23 Youzheng Street, Harbin, 150001 Heilongjiang Province China; 2 Department of General Surgery, Mulan Country People’s Hospital, Harbin, 150001 China

**Keywords:** DLEU1, Colorectal cancer, Progression, SMARCA1, KPNA3

## Abstract

**Background:**

Accumulating evidences show that long noncoding RNAs (lncRNA) play essential roles in the development and progression of various malignancies. However, their functions remains poorly understood and many lncRNAs have not been defined in colorectal cancer (CRC). In this study, we investigated the role of DLEU1 in CRC.

**Methods:**

Quantitative real-time PCR was used to detect the expression of DLEU1 and survival analysis was adopted to explore the association between DLEU1 expression and the prognosis of CRC patients. CRC cells were stably transfected with lentivirus approach and cell proliferation, migration, invasion and cell apoptosis, as well as tumorigenesis in nude mice were performed to assess the effects of DLEU1 in BCa. Biotin-coupled probe pull down assay, RNA immunoprecipitation and Fluorescence in situ hybridization assays were conducted to confirm the relationship between DLEU1 and SMARCA1.

**Results:**

Here we revealed that DLEU1 was crucial for activation of KPNA3 by recruiting SMARCA1, an essential subunit of the NURF chromatin remodeling complex, in CRC. DLEU1 was indispensible for the deposition of SMARCA1 at the promoter of KPNA3 gene. Increased expression of DLEU1 and KPNA3 was observed in human CRC tissues. And higher expression of DLEU1 or KPNA3 in patients indicates lower survival rate and poorer prognosis. DLEU1 knockdown remarkably inhibited CRC cell proliferation, migration and invasion in vitro and in vivo while overexpressing KPNA3 in the meantime reversed it.

**Conclusions:**

Our results identify DLEU1 as a key regulator by a novel DLEU1/SMARCA1/KPNA3 axis in CRC development and progression, which may provide a potential biomarker and therapeutic target for the management of CRC.

**Electronic supplementary material:**

The online version of this article (10.1186/s12943-018-0873-2) contains supplementary material, which is available to authorized users.

## Background

Colorectal cancer (CRC) is one of the leading causes and gives rise to large amounts of cancer-related deaths around the word every year [1, 2]. Currently, surgery and chemotherapy are the most common methods for CRC treatment [3]. Although some advances have been achieved on CRC treatment over recent decades, the overall survival rate of patients with advanced or metastatic CRC is still below 50% [4, 5]. The main cause is the increasing resistance to many anti-cancer agents in most CRC patients [6]. The therapeutic efficacy becomes disappointing. Accumulating studies have showed that some important genes regulate CRC development, such as APC and KRAS [7, 8]. However, the molecular mechanism that controls CRC development and progression still remains largely unknown. Therefore, to develop novel and effective approaches for CRC therapy, it is very necessary to define the molecular mechanism of CRC tumorigenesis.

Recent evidence demonstrates that nearly 98% of the genome transcripts in human are noncoding RNAs (ncRNA) [9, 10], among which long noncoding RNAs (lncRNAs) are transcripts of longer than 200 nucleotides and have no protein coding potential [11, 12]. More and more reports showed that lncRNAs have many kinds of biological functions involved in embryo development, immunoregulation, and tumor development [13–15]. Aberrant expression of lncRNAs is closely related with human diseases, especially in cancers [16, 17]. For example, long noncoding RNA PVT1 is up-regulated in hepatocellular carcinoma, nonsmall cell lung cancer, osteosarcoma, esophageal squamous cell carcinoma, cervical cancer, breast cancer and so on [18–23]. Furthermore, lncRNAs may control the resistance of tumor cells to drug. For instance, BCAR4 enhances cisplatin resistance in gastric cancer patients [24]. In colorectal cancer, many lncRNAs, including a large number of uncharacterized lncRNAs, are also abnormally expressed [25]. We showed that DLEU1 was up-regulated in CRC tissues compared to normal tissues. Previous research demonstrated that DLEU1 promotes ovarian carcinoma and gastric cancer development [26, 27]. Nevertheless, the roles of DLEU1 in other tumors including colorectal cancer remain elusive.

In this study, we found that DLEU1 was up-regulated in CRC tissues. Furthermore, overexpression of DLEU1 promoted CRC cell proliferation, migration and invasion in vitro and in vivo. In terms of mechanism, we found that DLEU1 co-localized with SMARCA1 in colorectal cancer cells. And DLEU1 is indispensible for the deposition of SMARCA1 at the promoter of KPNA3 gene. Collectively, DLEU1 recruited SMARCA1 to epigenetically activate downstream gene KPNA3, thereby promoting proliferation and migration in colorectal cancer. Therefore, our results propose a model for DLEU1-mediated cell proliferation in CRC.

## Methods

### Patient samples

100 pairs of CRC tissues and adjacent non-tumor tissues were obtained from The First Affiliated Hospital of Harbin Medical University. Three pathologists evaluated all specimens according to the World Health Organization (WHO) guidelines and the pTNM Union for International Cancer Control (UICC) pathological staging criteria. The samples were frozen in liquid nitrogen and stored at − 80 °C until use. Informed consent was obtained from all patients. The protocol was approved by The First Affiliated Hospital of Harbin Medical University. All methods involving human patients were performed in accordance with the relevant guidelines and regulations of The First Affiliated Hospital of Harbin Medical University.

### Cell lines and cell culture

The human colon cell lines (CCD18-Co, FHC and HCoEpiC) and human colorectal cancer cell lines (HCT116, HT29, SW480, SW620, DLD-1, LoVo, HCT8, RKO, and CaCo2) were purchased from the American Type Culture Collection (ATCC) and cultured according to their instructions. Cells were incubated at 37 °C in a humidified atmosphere with 5% CO_2_.

### Cell transfection

DLEU1, SMARCA1 and KPNA3 were cloned into pCDNA3 plasmid. shRNAs were synthetized by invitrogen and cloned into pGPH1/Neo (GenePharma, Shanghai, China) as described before [28]. Transfection was conducted with Lipofectamine 3000 (Invitrogen, Carlsbad, CA, USA) and stably DLEU1-silenced cell lines were screened out as previously described [28]. The shRNA sequences are as follows: shDLEU1: 5′-CACTTAAGCCTCGGAACAA-3′; shSMARCA1: 5′-TTGCCAGTTCCAGTGTATT-3′; shKPNA3: 5′-GTCTCAGTCACTTTGCAGT-3′.

### Antibodies

Anti-PCNA (13110), anti-MMP2 (87809), anti-TWIST (46702), anti-SMARCA1 (12483), anti-GAPDH (5014) and anti-CYCLIN D1 (2922) were purchased from Cell Signaling Technology. Anti-KPNA3 (HPA046852) was from Sigma.

### Apoptosis analysis

Cell apoptosis were analyzed by flow cytometry (FACScan; BD Biosciences) using CellQuest software (BD Biosciences).

### Tumor formation assay in vivo

The 6-week-old male athymic BALB/c nude mice were maintained under specific pathogen-free conditions and manipulated according to protocols approved by the Medical Experimental Animal Care Commission at The First Affiliated Hospital of Harbin Medical University. A volume of 0.1 ml of 4×10^6^ suspended cells was respectively subcutaneously injected into the posterior flank of each mouse. Tumor volumes and weight was measured at indicative time points.

### MTT assay and clone formation

MTT assay and clone formation were used for evaluated cell viability and proliferation. Cell proliferation was documented following the manufacturer’s protocol every 24 h. For the colony formation assay, cells were seeded in a fresh six-well plate and maintained in media containing 10% FBS, replacing the medium every 4 days. After 14 days, methanol and stained with 0.1% crystal violet (Sigma-Aldrich) fixed cells and count clones.

### In vitro migration and invasion assay

In the transwell migration assay, 5×10^4^ cells were placed in the top chamber of each insert (Millipore, Billerica, MA) with an uncoated membrane. For the invasion assay, 8×10^4^ cells were placed in the upper chamber of each insert coated with 100 μl Matrigel (BD Biosciences, MA) to form a matrix barrier. For both assays, cells were trypsinized and resuspended in 200 μl DMEM, and 500 μl DMEM supplemented with 10% FBS was added to the lower chamber. After incubation at 37 °C, any cells remaining in the top chamber or on the upper membrane of the inserts were carefully removed. After fixation and staining in a dye solution containing 0.1% crystal violet, the cells adhering to the lower membrane of the inserts were counted and imaged with an IX71 inverted microscope (Olympus Corp., Tokyo, Japan).

### Real-time quantitative PCR

Total RNAs were extracted with TRIzol according to the manufacturer’s protocol. Then cDNA was synthesized with the M-MLV reverse transcriptase (Promega). Then mRNA transcripts were analyzed with ABI 7300 qPCR system using specific primer pairs. Relative expression levels were calculated and normalized to endogenous *GAPDH* for mRNA and U6 for DLEU1. The primer sequence information is available if requested.

### Northern blot

Total RNA was extracted from sample cells with TRIzol. 10 μg RNA from each sample was subjected to formaldehyde-denaturing agarose electrophoresis followed by transferring to positively charged NC film with 20 × SSC buffer. Membrane was UV cross-linked and incubated with hybrid buffer for a 2 h prehybridization, followed by incubation with biotin-labeled RNA probes. Biotin signals were detected with HRP-conjugated streptavidin according to the manufacturer’s instruction.

### In situ hybridization

Samples were fixed and embedded with paraffin. Then sample sections were incubated in graded alcohols and incubated in 3% hydrogen peroxide (H_2_O_2_) for 30 min. Biotin-conjugated probes and streptavidin-HRP conjugate were used for ISH. The samples were finally stained with haematoxylin. The probe sequences for DLEU1 were as follows: 5′-ACGATGATTCTGCGCATGTG-3′ and 5′-CTGGTAGCTATAAGACGACC-3′.

### DNA FISH

Cells were fixed with 4% PFA containing 10% acetic acid for 15 min at room temperature, followed by replacement with 70% ethanol at − 20 °C. Cells were then incubated in buffer containing 100 mM Tris-HCl (pH 7.5), 150 mM NaCl, followed by cytoplasm digestion in 0.01% pepsin/0.01 N HCl for 3 min at 37 °C. Cells were further fixed in 3.7% PFA and replaced with ethanol to a final concentration of 100%. Cells were air dried and washed with 2×SSC, followed by blocking with buffer containing 100 mM Tris-HCl (pH 7.5), 150 mM NaCl, 0.05% Tween 20, 3% BSA for 20 min. Cells were then denatured in 70% formamide/2×SSC, and incubated with fluorescence-labeled DNA probes overnight. Cells were counterstained with DAPI for nucleus post washing with PBS.

### RNA pulldown

Biotin-labeled RNAs were transcribed in vitro with the Biotin RNA Labeling Mix (Roche Diagnostics) and T7 RNA polymerase (Roche Diagnostics), treated with RNase-free DNase I (Roche), and purified with an RNeasy Mini Kit (Qiagen, Valencia, CA). Next, whole-cell lysates were incubated with 3 μg of purified biotinylated transcripts for 1 h at 25 °C. Complexes were isolated with streptavidin agarose beads (Invitrogen). The beads were washed briefly three times and boiled in sodium dodecyl sulfate (SDS) buffer, and the retrieved protein was detected by western blot or mass spectrum.

### RNA immunoprecipitation (RIP)

We performed RNA immunoprecipitation (RIP) experiments using the Magna RIP™RNA-Binding Protein Immunoprecipitation Kit (Millipore, USA) according to the manufacturer’s instructions. The co-precipitated RNAs were detected by reverse-transcription PCR. The total RNAs were the input controls.

### Chromatin immunoprecipitation (ChIP)

We conducted ChIP using the EZ ChIP™Chromatin Immunoprecipitation Kit for cell line samples (Millipore, Bedford, MA). Briefly, we sonicated the crosslinked chromatin DNA into 200- to 500-bp fragments. The chromatin was then immunoprecipitated using primary antibodies. Normal IgG was used as the negative control. Quantification of the immunoprecipitated DNA was performed using qPCR with SYBR Green Mix (Takara).

### Statistical analysis

All statistical analyses were performed using the Statistical Package for the Social Sciences version 20.0 software (SPSS Inc., Chicago, IL, USA). Survival curves were calculated using the Kaplan-Meier method and were analyzed using the log-rank test. For comparisons, one-way analyses of variance and two-tailed Student’s t-tests were performed, as appropriate. *P* < 0.05 was considered statistically significant.

## Results

### DLEU1 expression is up-regulated in human CRC tissues

**Fig. 1:**
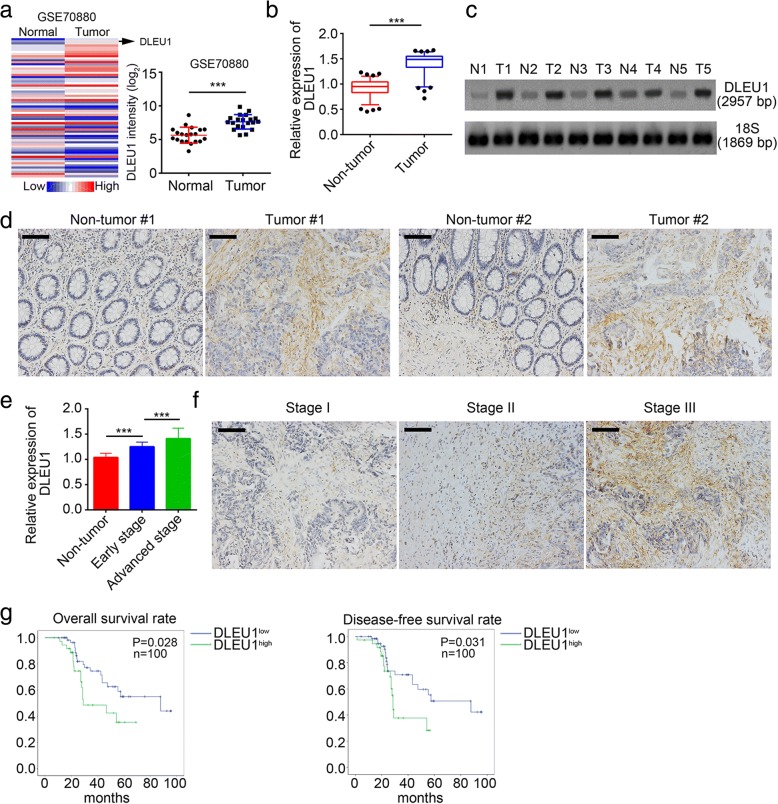
DLEU1 expression is up-regulated in human CRC tissues*.* **a** According to an online database (GSE70880), DLEU1 showed higher expression level in CRC tissues compared to non-tumor tissues. **b** RNAs were extracted from CRC samples and non-tumor tissues, and then the expression of DLEU1 was analyzed by RT-qPCR. **c** The expression levels of DLEU1 were measured by Northern blot in pairs of CRC samples. DLEU1 and 18S probes were biotin-labeled. **d** DLEU1 expression was checked by in situ hybridization in CRC samples. Scale bar, 100μm. **e** DLEU1 expression levels were highest in CRC samples with advanced stage. 100 CRC samples collected were grouped into early stage and advanced stage based on clinical characteristics. **f** DLEU1 showed highest expression levels in stage III CRC samples. 100 CRC samples were grouped into stage I, stage II and stage III based on clinical characteristics. Scale bar, 100μm. **g** 100 CRC samples were divided into two groups according to DLEU1 expression and Kaplan–Meier survival analysis was conducted. Patients with higher DLEU1 expression possessed lower survival rates. ***P<0.001. All data were collected from three independent experiments

To understand the role of lncRNAs in colorectal cancer, we first analyzed differentially expressed lncRNAs between colorectal cancer tissues and normal tissues according to a microarray data (GSE70880) [29]. We found that DLEU1 was one of the most up-regulated lncRNAs in CRC tissues according to this dataset (Fig. 1a). Next, we used RT-qPCR to analyze DLEU1 expression in 100 pairs of CRC samples and adjacent histologically normal tissues. We found that DLEU1 was remarkably up-regulated in CRC tissues compared to non-tumor tissues (Fig. 1b). Furthermore, we performed Northern blot and in situ hybridization (ISH). We found that CRC samples displayed higher expression of DLEU1 than non-tumor tissues (Fig. 1c, d). Then we checked the expression of DLEU1 in early stage and advanced CRC samples by RT-qPCR. The expression of DLEU1 was highest in advanced CRC samples (Fig. 1e). Besides, we found that the expression of DLEU1 in CRC was positively correlated with tumor clinical stage through ISH. As shown, DLEU1 expression was higher in Stage II and Stage III tissues than in Stage I tissues (Fig. 1f). Next, we classified the 100 colorectal cancer samples into two groups according to DLEU1 expression. We then analyzed the relationship between DLEU1 expression and patients’ survival rate. We found that CRC patients with higher DLEU1 expression possessed lower survival rates (Fig. 1g). Summarily, DLEU1 was up-regulated in colorectal cancer and may serve as a biomarker for CRC prognosis.

DLEU1 knockdown inhibits cell proliferation, migration and invasion in CRC.

**Fig. 2:**
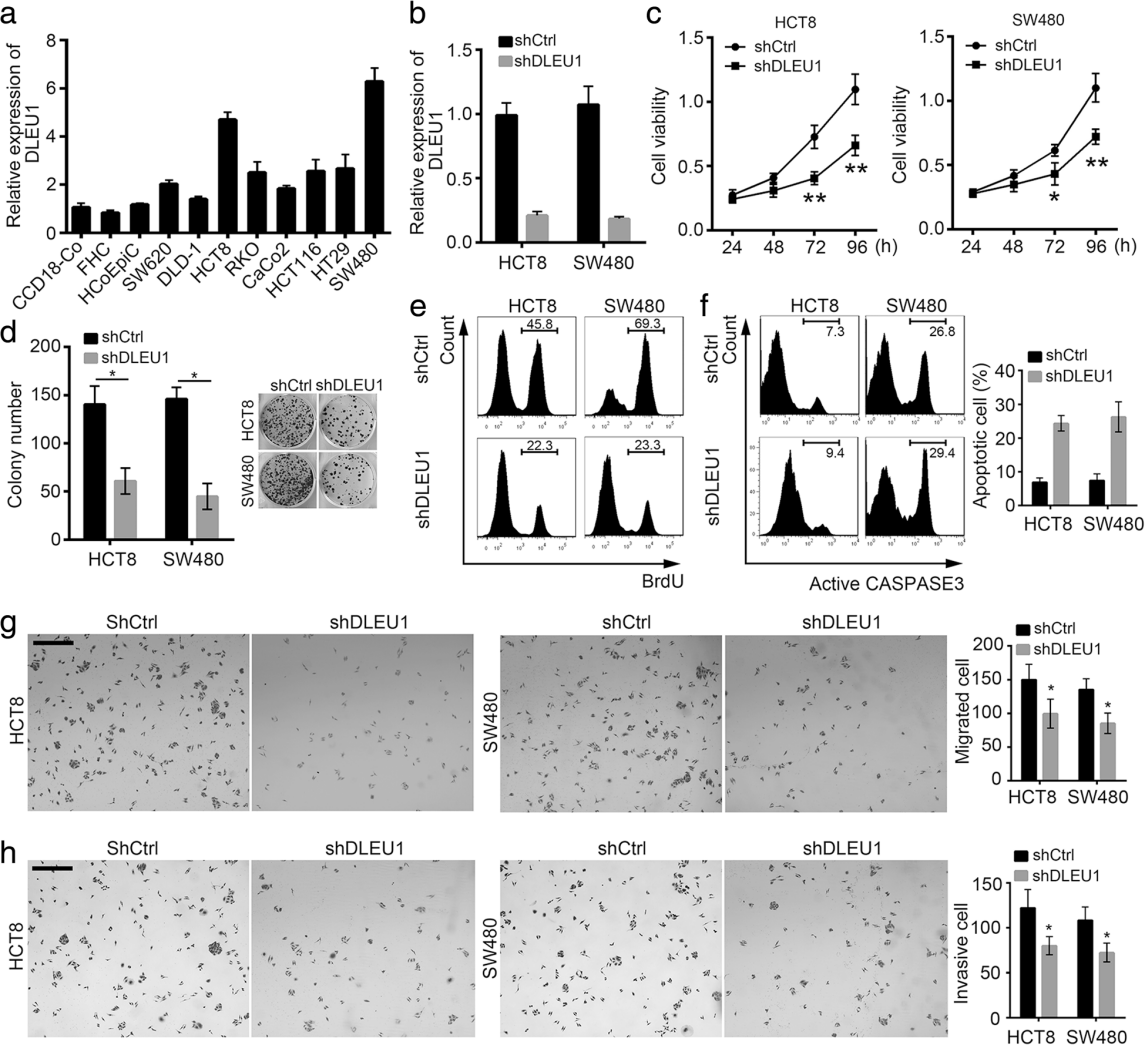
DLEU1 knockdown inhibits cell proliferation, migration and invasion in CRC. **a** Total RNAs were extracted from CRC cell lines (SW620, DLD-1, HCT8, RKO, CaCo2, HCT116, HT29 and SW480) and human colon cell line (CCD18-Co, FHC and HCoEpiC). Then the expression of DLEU1 was analyzed by RT-qPCR. **b** DLEU1 was successfully knocked down in HCT8 and SW480 cells. **c** DLEU1 knockdown dramatically inhibited cell proliferation. MTT assays were performed with shCtrl and shDLEU1 HCT8 or SW480 cells. Cell viabilities were measured at different time points. **d** DLEU1 knockdown remarkably impaired the ability of colony formation. **e** DLEU1 depletion decreased cell division. shCtrl and shDLEU1 HCT8 or SW480 cells were cultured in the presence of BrdU for 6 h. Then cells were collected and the incorporation of BrdU was analyzed by FACS. **f** DLEU1 knockdown greatly promoted cell death in HCT8 and SW480 cells. Cell apoptosis was evaluated by staining with active CASPASE 3 and analysis with FACS. **g, h** DLEU1 depletion inhibited cell migration and invasion. Scale bar, 50 μm. *P<0.05 and **P < 0.01. All data were collected from three independent experiments

To define the effect of DLEU1 on CRC cells, we analyzed the endogenous expression of DLEU1 in various CRC cell lines by RT-qPCR. We found that CRC cells had higher expression of DLEU1 than human colon fibroblast cell line (CCD18-Co) (Fig. 2a). Because DLEU1 expression was the highest in HCT8 and SW480 cells, we chose these two cell lines for following experiments. We knocked down DLEU1 in HCT8 and SW480 cells effectively (Fig. 2b). We performed MTT assays and found that DLEU1 knockdown significantly decreased the proliferation abilities of HCT8 and SW480 cells compared to respective controls (Fig. 2c). Besides, colony formation assays showed that DLEU1 knockdown inhibited colony formation (Fig. 2d). To further evaluate cell proliferation, we conducted BrdU incorporation assays. As shown, deficiency of DLEU1 decreased BrdU incorporation in HCT8 and SW480 cells (Fig. 2e). Moreover, we found that DLEU1 depletion markedly promoted cell death as more active CASPASE3^+^ cells appeared after DLEU1 knockdown (Fig. 2f). Finally, we analyzed the effect of DLEU1 on migration and invasion. As shown, after DLEU1 silence, the abilities of cell migration and invasion were remarkably impaired (Fig. 2g, h). Taken together, above results indicated that DLEU1 possessed a key role on regulating malignant behaviors of CRC.

DLEU1 overexpression promoted CRC cell proliferation, migration and invasion.

**Fig. 3:**
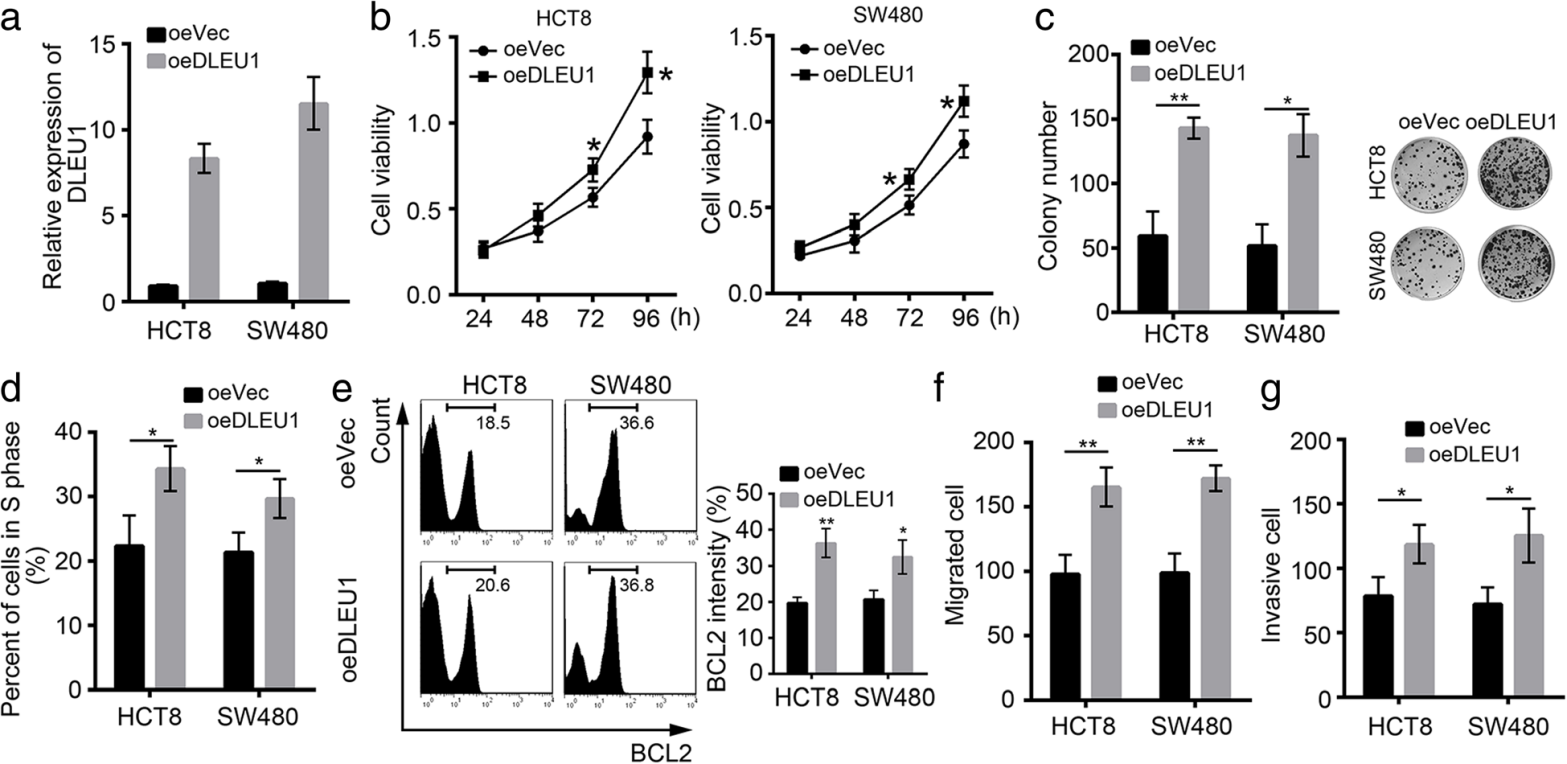
DLEU1 overexpression promoted CRC cell proliferation, migration and invasion. **a** DLEU1 was overexpressed in HCT8 and SW480 cells. **b** DLEU1 overexpression promoted cell proliferation as shown by MTT assays. **c** overexpressing DLEU1 increased the ability of colony formation by HCT8 and SW480 cells. **d** More DLEU1-overexpressing cells entered into S phase as shown by FACS. **e** DLEU1 overexpression promoted BCL2 expression in HCT8 and SW480 cells. **f, g** DLEU1 overexpression promoted cell migration and invasion. *P<0.05 and **P<0.01. All data presented are shown as means ± SD collected from three independent experiments

To further confirm the function of DLEU1 in CRC, we overexpressed DLEU1 in HCT8 and SW480 cells (Fig. 3a). Overexpression of DLEU1 promoted cell proliferation as shown by MTT assays (Fig. 3b). Moreover, DLEU1 overexpression promoted colony formation by HCT8 and SW480 cells (Fig. 3c). In consistence, more DLEU1-overexpressing HCT8 and SW480 cells entered into S phase (Fig. 3d). Then we evaluated cell apoptosis by FACS. We found that the intensity of BCL2 was increased in HCT8 and SW480 cells after DLEU1 overexpression (Fig. 3e), which indicated that overexpressing DLEU1 enhanced cell survival. Additionally, more CRC cells migrated and invaded after ectopic expression of DLEU1 (Fig. 3f, g). Moreover, we also found that overexpression of DLEU1 promoted the proliferation, migration and invasion of HCT116 and SW620 cells (Additional file 1: Figure S1a-d).

### DLEU1 depletion delayed tumor growth in vivo

**Fig. 4:**
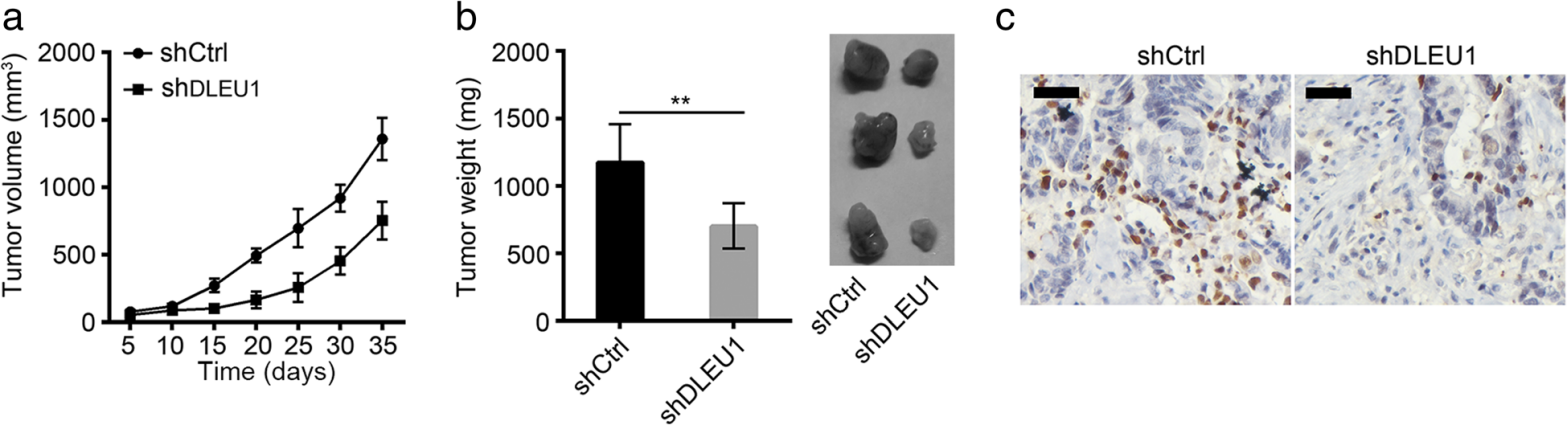
DLEU1 depletion delayed tumor growth in vivo. **a** 4×10^6^ shCtrl or shDLEU1 cells were injected into 6-week-old nude mice subcutaneously. Then tumor volumes were monitored at different time points. DLEU1 knockdown markedly delayed tumor growth in mice. **b** Tumor weights were measured 5 weeks later post injection. DLEU1 knockdown dramatically decreased tumor weight. **c** Ki67 expression was measured in formed tumor tissues by IHC. Scale bar, 20 μm. **P<0.01. All data presented are shown as means ± SD collected from three independent experiments

To further demonstrate the role of DLEU1 in vivo, we injected shCtrl and shDLEU1 HCT8 cells into 6-week-old nude mice subcutaneously. We then measured the tumor volumes at indicative time points. We found that DLEU1 knockdown significantly delayed tumor growth in vivo (Fig. 4a). And 5 weeks later, we checked the weights of formed tumors. We also found that tumor tissues derived shDLEU1 HCT8 cells were lighter (Fig. 4b). Moreover, the Ki67^+^ cells in control tumor tissues were more than in shDLEU1 tissues (Fig. 4c). Taken together, these results showed that DLEU1 suppressed tumor growth in vivo.

### DLEU1 interacts with SMARCA1 in CRC cells

**Fig. 5:**
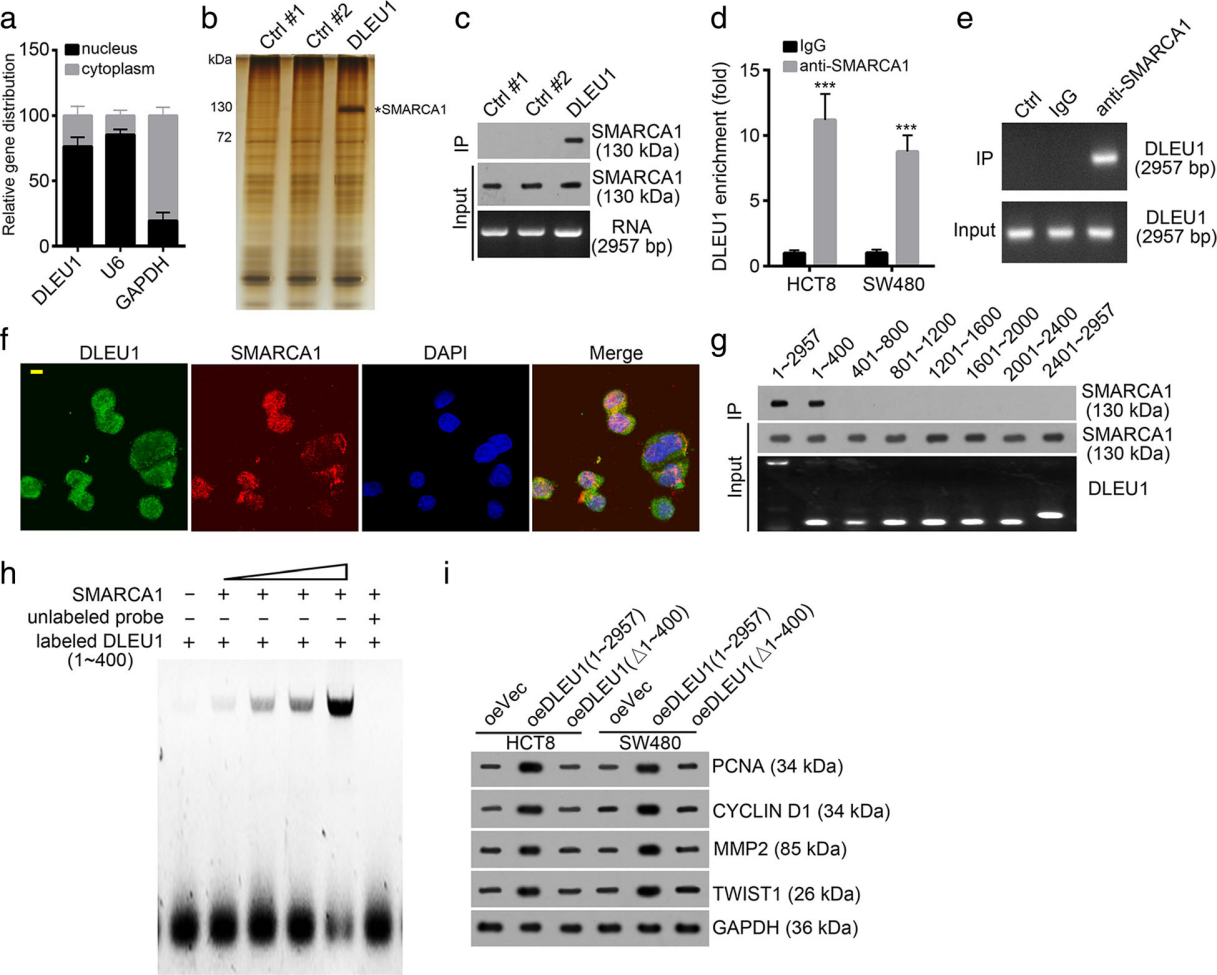
DLEU1 interacts with SMARCA1 in CRC cells. **a** The expression of DLEU1 in cytoplasm and nucleus of HCT8 cells was measured by qRT-PCR. U6 serves as a nuclear control. GAPDH serves as a cytoplasmic control. **b** SMARCA1 was a potential interactive candidate of DLEU1. Biotin-labeled DLEU1 and intron control were incubated with HCT8 cell lysates, and the enriched products were eluted and separated by SDS-PAGE electrophoresis and silver staining. The differential band appearing in DLEU1 lane was analyzed by mass spectrum. **c** DLEU1 associated with SMARCA1 as shown by RNA pulldown and Western blot. Biotin-labeled DLEU1 and intron control were added into HCT8 cell lysates, and pulldown assays were performed. **d** DLEU1 was enriched by SMARCA1 in HCT8 and SW480 cell lysates. **e** SMARCA1 enriched DLEU1 in HCT8 cell lysates. SMARCA1 antibody was added into cell lysates and enriched RNAs were isolated. Then enriched DLEU1 was analyzed by PCR. **f** DLEU1 co-localized with SMARCA1 in HCT8 cells as shown by RNA FISH. Green, DLEU1; Red, SMARCA1; Blue, DAPI. Scale bar, 10 μm. **g** the region of nt 1~ 400 in DLEU1 was important for the interaction with SMARCA1. **h** DLEU1 (nt 1~ 400) associated with SMARCA1 directly as shown by RNA EMSA assays. **i** The region of nt 700~ 1050 is indispensable for the function of DLEU1 in colorectal cancer. Overexpression of DLEU1 with deletion of nt 1~ 400 cannot promoted proliferation and metastasis in CC. ***P<0.001. All data presented are shown as means ± SD collected from three independent experiments

Previous study demonstrated that lncRNAs can interact with proteins to regulate gene expression [30]. We also observed that DLEU1 was mainly located in nucleus of HCT8 cells (Fig. 5a). Hence, we performed RNA pulldown and mass spectrum to identify potential interactive proteins. We, firstly, obtained biotin-labeled DLEU1 and two intron controls by T7 transcription in vitro. Then we added biotin-labeled DLEU1 or controls into HCT8 cell lysates and incubated with Streptavidin C1 beads. Then the enrichment products were eluted and resolved with SDS-PAGE and silver staining. The differential band that appeared in the DLEU1 lane was cut for identification by mass spectrum. SMARCA1, an essential subunit of chromatin remodeling NURF complex, was identified as a potential candidate (Fig. 5b). Then we confirmed the interaction between DLEU1 and SMARCA1 by RNA pulldown assays (Fig. 5c). Moreover, RNA IP assays showed that SMARCA1 antibody also enriched DLEU1 in HCT8 and SW480 cells (Fig. 5d). We further confirmed it by PCR and DNA gel electrophoresis (Fig. 5e). Furthermore, we conducted RNA fluorescence in situ hybridization (RNA-FISH) and found that DLEU1 was co-localized with SMARCA1 in HCT8 cells (Fig. 5f). The length of DLEU1 was 2957 nucleotides. To search the interactive domain in DLEU1 with SMARCA1, we constructed 7 truncations and performed RNA pulldown assays. We found that the region of nt 1~ 400 was essential for binding to SMARCA1 (Fig. 5g). What’s more, we performed RNA electrophoretic mobility shift assay (RNA EMSA). We found that DLEU1 (nt 1~ 400) directly bound to SMARCA1 (Fig. 5h). We wonder whether this domain (nt 1~ 400) was vital for its function. We overexpressed DLEU1 full-length and truncation (deletion of nt 1~ 400) in HCT8 and SW480 cells. However, only DLEU1 full-length promoted the expression of PCNA, CYCLIN D1, MMP2 and TWIST1 in CRC cells (Fig. 5i), which suggested the region of nt 1~ 400 is essential for the function of DLEU1. Altogether, DLEU1 directly bound to SMARCA1 relying on the region of nt 1~ 400 in CRC.

### DLEU1 promotes KPNA3 expression by recruiting SMARCA1 in CRC

**Fig. 6:**
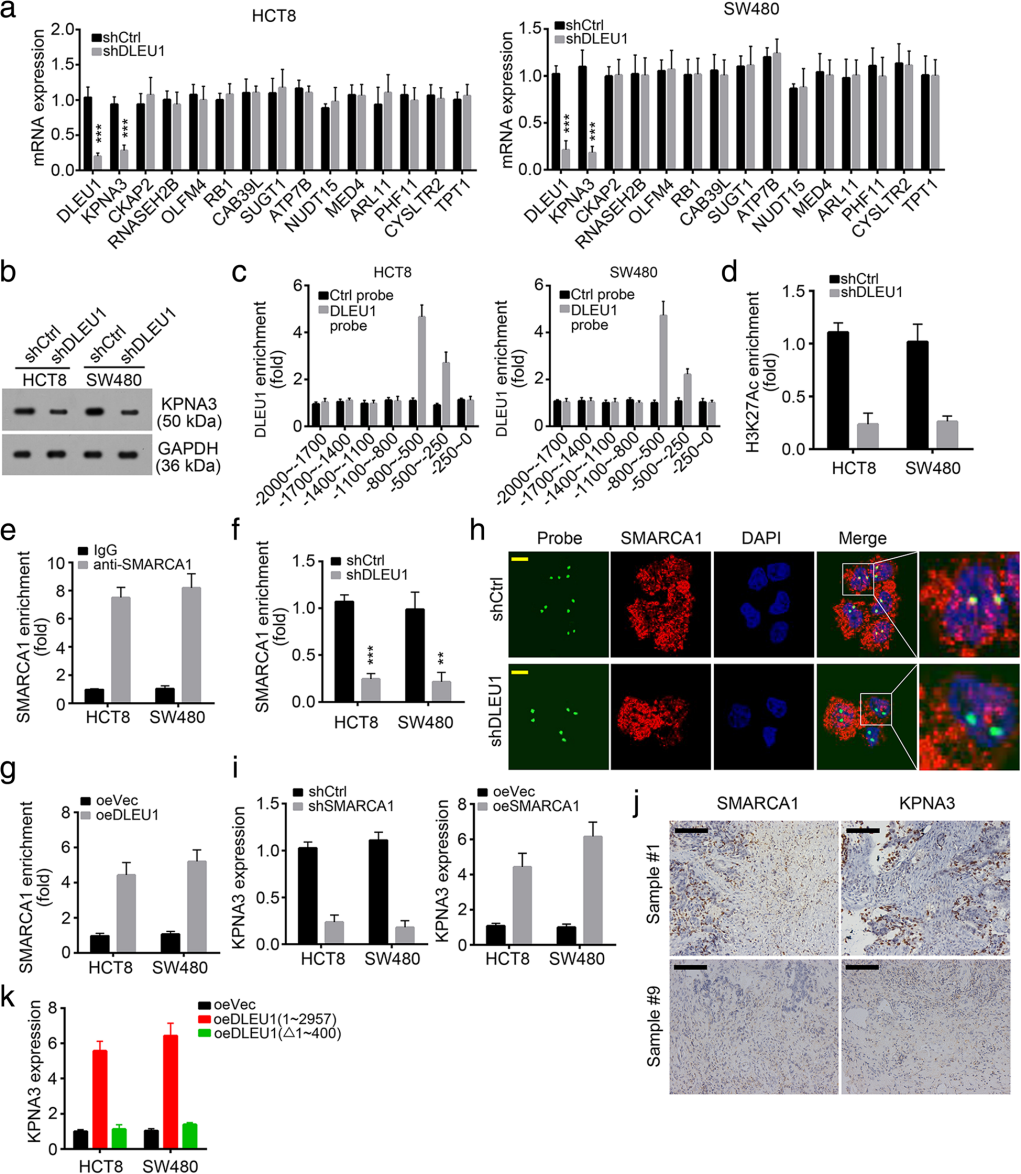
DLEU1 promotes KPNA3 expression by recruiting SMARCA1 in CRC. **a** DLEU1 knockdown impaired the expression of KPNA3 in HCT8 and SW480 cells. The expression levels of neighboring genes of DLEU1 were measured by RT-qPCR after DLEU1 knockdwon. **b** DLEU1 depletion decreased the protein levels of KPNA3 in HCT8 and SW480 cells. **c** DLEU1 enriched on the promoter (− 800 bp~ − 250 bp from TSS) of KPNA3 in HCT8 and SW480 cells. **d** DLEU1 knockdown inhibited the enrichment of H3K27Ac on KPNA3 promoter in HCT8 and SW480 cells. **e** SMARCA1 enriched on KPNA3 promoter in HCT8 and SW480 cells. **f** DLEU1 knockdown impaired the enrichment of SMARCA1 on KPNA3 promoter in HCT8 and SW480 cells. **g** DLEU1 overexpression promoted the enrichment of SMARCA1 on KPNA3 promoter in HCT8 and SW480 cells. **h** SMARCA1 co-localized with KPNA3 promoter in HCT8 cells while DLEU1 knockdown abrogated it. Green, KPNA3 promoter probe; Red, SMARCA1; Blue, DAPI. Scale bar, 10 μm. **i** SMARCA1 silence decreased the expression of KPNA3 in HCT8 and SW480 cells while SMARCA1 overexpression up-regulated that of KPNA3. **j** Higher expression of KPNA3 in SMARCA1 highly expressed CC samples. Scale bar, 100μm. **k** Overexpressing DLEU1 promoted KPNA3 expression while deletion of nt 1~ 400 abrogated it in HCT8 and SW480 cells. **P<0.01 and ***P<0.001. All data presented are shown as means ± SD collected from three independent experiments

Many evidences proved that lncRNAs may regulate the expression of their neighbor genes [31]. To define the downstream target gene of DLEU1 in CRC cells, we analyzed the expression of the neighbor genes of DLEU1 by RT-qPCR. We found that the expression of KPNA3 was significantly down-regulated after DLEU1 knockdown in HCT8 and SW480 cells (Fig. 6a, b). Then we conducted chromatin isolation by RNA purification (CHIRP) assays. As shown, KPNA3 promoter (− 800~-250 bp from transcription start site) was enriched by DLEU1 in HCT8 and SW480 cells (Fig. 6c). Moreover, DLEU1 knockdown really decreased the enrichment of H3K27Ac, a histone modification indicating transcriptional activation (Fig. 6d). Our above data showed that DLEU1 associated with SMARCA1. To define whether SMARCA1 also regulated KPNA3 expression, we performed chromatin immunoprecipitation (ChIP) assays. We found that SMARCA1 enriched at the same region of KPNA3 promoter as DLEU1 in HCT8 and SW480 cells (Fig. 6e). Nevertheless, DLEU1 knockdown impaired the enrichment of SMARCA1 at KPNA3 promoter (Fig. 6f), and vice versa (Fig. 6g). The results by DNA-FISH also demonstrated that DLEU1 depletion abrogated the co-localization between SMARCA1 and KPNA3 promoter in HCT8 cells (Fig. 6h). Furthermore, we found that SMARCA1 knockdown inhibited the mRNA levels of KPNA3 in HCT8 and SW480 cells, and vice versa (Fig. 6i). Moreover, we found that higher expression of SMARCA1 in human CRC samples indicated higher expression of KPNA3 (Fig. 6j). Our data showed that DLEU1 (nt 1~ 400) is essential for its association with SMARCA1. To confirm whether DLEU1 (nt 1~ 400) is indispensible to regulate KPNA3 expression. We found that overexpressing DLEU1 but not truncation (deletion of nt 1~ 400) promoted KPNA3 expression in HCT8 and SW480 cells (Fig. 6k). In summary, our results revealed that DLEU1 recruited SMARCA1 to KPNA3 promoter and promoted KPNA3 transcription. And DLEU1 (nt 1~ 400) is indispensible in this process.

### DLEU1 promotes CRC cell proliferation, migration and invasion by activation of KPNA3

**Fig. 7:**
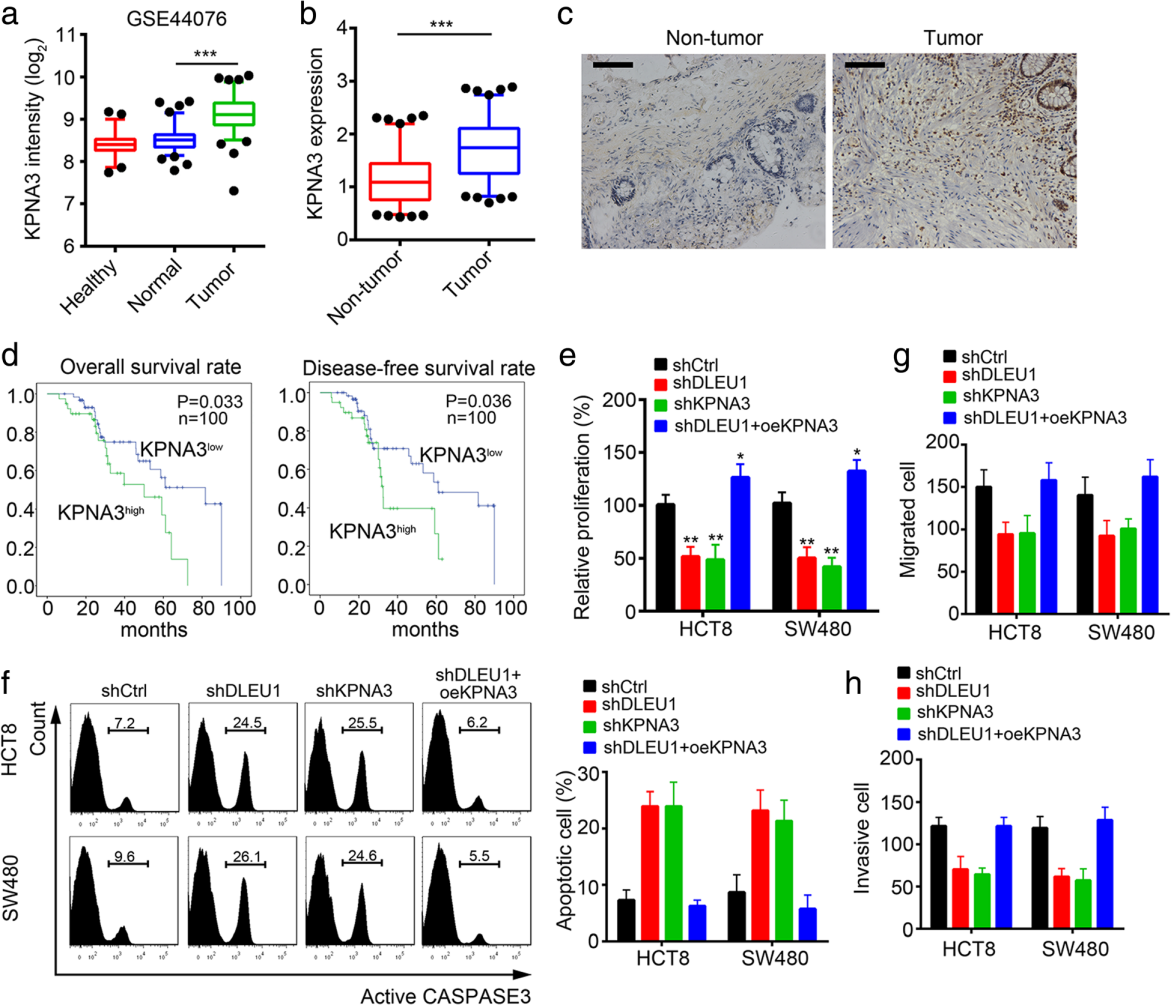
DLEU1 promotes CRC cell proliferation, migration and invasion by activation of KPNA3. **a** KPNA3 was up-regulated in CRC tissues according to a database (GSE44076). **b** KPNA3 was highly expressed in CRC samples. Total RNAs were extracted from CRC tissues and non-tumor tissues and the mRNA levels of KPNA3 were analyzed by RT-qPCR. **c** The expression of KPNA3 in CRC tissues was checked by IHC. Scale bar, 100μm. **d** 100 CRC samples were divided into two groups according to KPNA3 expression and Kaplan–Meier survival analysis was conducted. Patients with higher KPNA3 expression possessed lower survival rates. **e** Knockdown of DLEU1 or KPNA3 inhibited cell proliferation in HCT8 and SW480 cells while KPNA3 overexpression promoted it. **f** DLEU1 or KPNA3 depletion increased cell apoptosis in HCT8 and SW480 cells while KPNA3 overexpression reversed it. **g**, **h** depletion of DLEU1 or KPNA3 impaired cell migration and invasion in HCT8 and SW480 cells while KPNA3 ectopic expression inhibited it. **P<0.01, *P<0.05 and ***P<0.001. All data presented are shown as means ± SD collected from three independent experiments

To further examine whether DLEU1 regulated CRC development and progression by activating KPNA3, we analyzed the expression of KPNA3 in CRC tissues and non-tumor tissues. We found that KPNA3 was up-regulated in CRC tissues according to the dataset (GSE44076) (Fig. 7a) [32]. KPNA3 was also up-regulated in the 100 CRC samples compared to non-tumor tissues (Fig. 7b). The results by immunohistochemistry (IHC) showed a similar trend (Fig. 7c). Moreover, Kaplan–Meier survival analysis indicated that higher expression of KPNA3 in patients with CRC stood for lower survival rates (Fig. 7d). Then we performed MTT assays. We found that knockdown of DLEU1 or KPNA3 inhibited cell proliferation while overexpressing KPNA3 reversed it in HCT8 and SW480 cells (Fig. 7e). Besides, knockdown of DLEU1 or KPNA3 promoted cell apoptosis while KPNA3 overexpression rescued it (Fig. 7f). In consistence, knockdown of DLEU1 or KPNA3 impaired cell migration and invasion while overexpressing KPNA3 reversed it (Fig. 7g, h). Collectively, our data demonstrated that DLEU1 regulated cell proliferation, apoptosis, migration and invasion by activation of KPNA3 in CRC.

## Discussion

Accumulating evidences have demonstrated the importance of lncRNAs in various human tumors, including CRC [33–35]. The expression of lncRNAs is often abnormal in human cancers [36]. Therefore, many lncRNAs are reported to serve as a biomarker for tumor diagnosis [37]. Seeking the key lncRNAs and understanding their functional mechanism are a matter of great significance for diagnosis, therapy and prognosis of different cancers. However, lncRNAs in CRC are still an emerging field, only a few lncRNAs have been defined in CRC and should be further explored as predictive biomarkers. In our study, we found that the expression of DLEU1 was remarkably up-regulated in CRC tissues and correlated with clinical severity.

Our data proved that DLEU1 knockdown significantly inhibited cell proliferation both in vitro and in vivo, whereas overexpressing DLEU1 promoted tumor growth. Depletion of DLEU1 led to decreased cell division. Fewer cells entered into S phase after DLEU1 knockdown. Accumulating evidences showed that lncRNAs regulate tumorigenesis and cancer progression by various mechanisms including epigenetic regulation and transcriptional regulation [38–40]. To reveal the underlying mechanism, we performed RNA pulldown and mass spectrum assays. We identified SMARCA1 as an interactive protein of DLEU1. SMARCA1 is an essential subunit of the chromatin remodeling NURF complex [41, 42]. NURF complex promotes target gene expression by remodeling chromatin accessibility. However, the role of SMARCA1 in CRC has not been explored. In our study, we found that DLEU1 interacted with SMARCA1 directly in CRC cells and regulated cancer cell proliferation.

Many evidences proved that lncRNAs may regulate the expression of their neighbor genes [31]. To define the downstream target gene of DLEU1 in CRC, we analyzed the expression of the neighbor genes of DLEU1 by RT-qPCR. We found that DLEU1 knockdown significantly down-regulated the expression of KPNA3. The function of KPNA3 remains elusive in CRC. In our study, we found that the expression of KPNA3 was significantly up-regulated in CRC tissues compared to non-tumor tissues. Our data demonstrated that DLEU1 and SMARCA1 deposited at the same promoter region of KPNA3. Moreover DLEU1 (nt 1~ 400) is indispensible to recruit SMARCA1 at KPNA3 promoter. DLEU1 and SMARCA1 cooperated to promote KPNA3 activation in CRC. Furthermore, KPNA3 knockdown remarkably inhibited cell proliferation, migration and invasion, and vice versa. Nevertheless, how KPNA3 exerts roles in CRC progression still requires to be further investigated. KPNA3 is a subunit of the nuclear pore complex (NPC) and involved in nuclear protein import [43]. KPNA3 might regulate protein transfer to promote CRC growth, metastasis and relapse.

## Conclusions

In conclusion, we had demonstrated that DLEU1 was highly expressed in CRC tissues and its up-regulation may predict poor prognosis. DLEU1 promoted CRC cell proliferation and tumorigenesis in vitro and in vivo. In addition, we defined the molecular mechanism by which DLEU1 contributes to CRC progression. Finally, these data provided new insights on how lncRNAs target chromatin-remodeling proteins to regulate gene expression.

## Additional file

Additional file 1: **Figure S1.** Overexpression of DLEU1 promotes CRC cell proliferation, migration and invasion. a The expression of DLEU1 was measured by qRT-PCR in HCT116 and SW620 cells transfected with DLEU1 ectopic expressing vector or control. b CCK8 assay was used for analysis of cell proliferation. c, d Transwell assay was utilized to determine cell migration and invasion. *P<0.05. All data were collected from three independent experiments. (DOCX 133 kb)

## Abbreviations

CRC: Colorectal cancer
DLEU1: Deleted in lymphocytic leukemia 1
IHC: Immunohistochemistry
ISH: In situ hybridization
lncRNA: Long noncoding RNA
qRT-PCR: Quantitative-Reverse transcription polymerase chain reaction
