# Supplementary material for: LncRNA DLEU1 contributes to colorectal cancer progression via activation of KPNA3
Source: Mol Cancer. 2018 Aug 11;17:118. doi: 10.1186/s12943-018-0873-2 (PMC6087004; doi:10.1186/s12943-018-0873-2)
Supplement: Supplementary file 1 — Figure S1. Overexpression of DLEU1 promotes CRC cell proliferation, migration and invasion. a The expression of DLEU1 was measured by qRT-PCR in HCT116 and SW620 cells transfected with DLEU1 ectopic expressing vector or control. b CCK8 assay was used for analysis of cell proliferation. c, d Transwell assay was utilized to determine cell migration and invasion. *P<0.05. All data were collected from three independent experiments. (DOCX 133 kb) [file 12943_2018_873_MOESM1_ESM.docx]

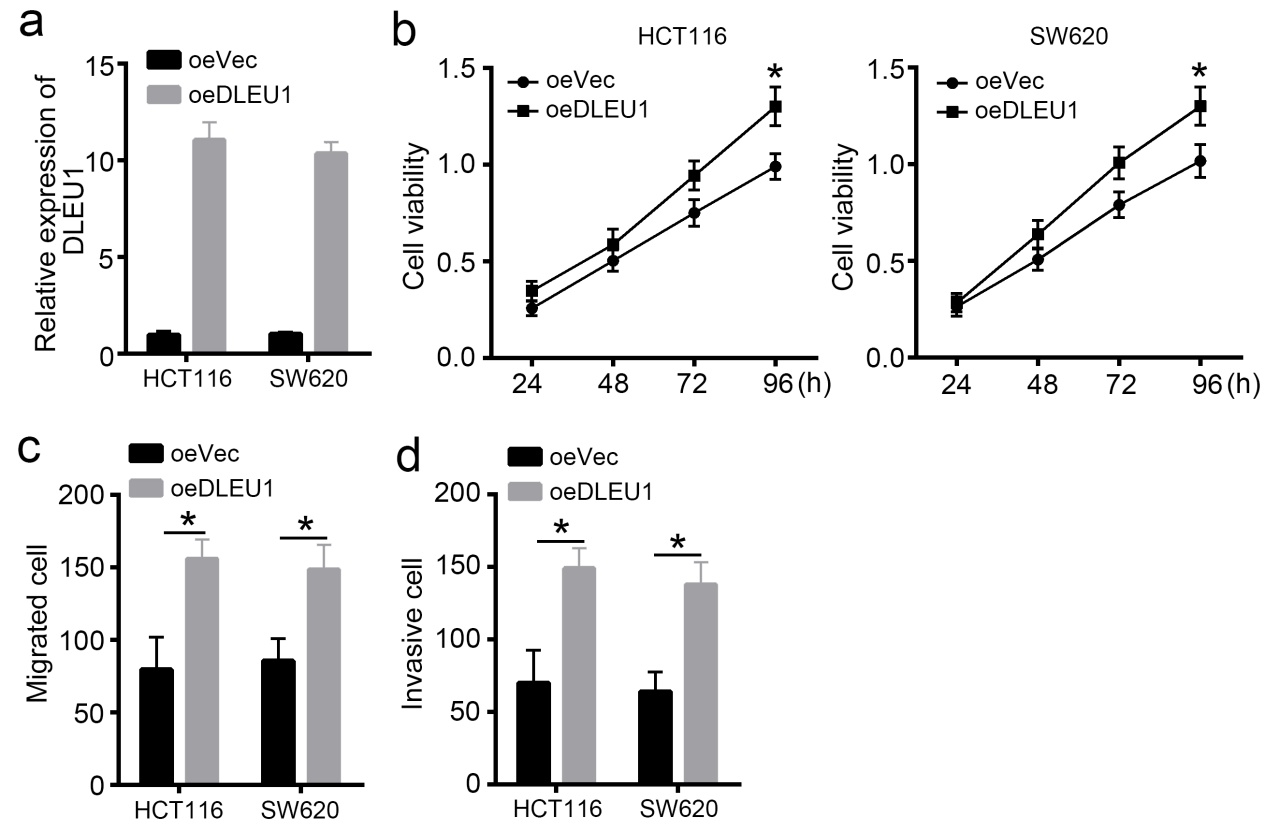


**Additional file 1: Figure S1 Overexpression of DLEU1 promotes CRC cell proliferation, migration and invasion.** **a** The expression of DLEU1 was measured by qRT-PCR in HCT116 and SW620 cells transfected with DLEU1 ectopic expressing vector or control. **b** CCK8 assay was used for analysis of cell proliferation. **c**, **d** Transwell assay was utilized to determine cell migration and invasion. **P*<0.05. All data were collected from three independent experiments.
